# Modern Techniques in Re-Irradiation for Locally Recurrent Rectal Cancer: A Systematic Review

**DOI:** 10.3390/cancers15194838

**Published:** 2023-10-03

**Authors:** Giovanna Mantello, Elena Galofaro, Silvia Bisello, Giuditta Chiloiro, Angela Romano, Luciana Caravatta, Maria Antonietta Gambacorta

**Affiliations:** 1 Radiotherapy Department, Azienda Ospedaliero Universitaria delle Marche, 60126 Ancona, Italy; giovanna.mantello@ospedaliriuniti.marche.it (G.M.); silvia.bisello@ospedaliriuniti.marche.it (S.B.); 2 Departments of Radiation Oncology, Fondazione Policlinico Universitario A.Gemelli IRCCS, 00168 Roma, Italy; giuditta.chiloiro@policlinicogemelli.it (G.C.); angela.romano1@guest.policlinicogemelli.it (A.R.); mariaantonietta.gambacorta@policlinicogemelli.it (M.A.G.); 3 Department of Radiation Oncology, SS Annunziata Hospital, 66100 Chieti, Italy; lcaravatta@hotmail.com

**Keywords:** recurrence, rectal cancer, re-irradiation, radiotherapy, review

## Abstract

**Simple Summary:**

Re-irradiation of locally recurrent rectal cancer presents challenges in terms of treatment options and outcomes. By conducting a systematic review focused on new technologies such as carbon ion radiotherapy, intensity-modulated photon radiotherapy, and stereotactic radiotherapy, we aimed to determine whether the new techniques have led to improvements in both outcomes and toxicities to enable clinicians and researchers to make informed decisions about incorporating new technologies into clinical practice and to identify avenues for further research.

**Abstract:**

Background: Radiotherapy (RT) plays an important role in the treatment of patients with previously irradiated locally recurrent rectal cancer (LRRC). Over the years, numerous technologies and different types of RT have emerged. The aim of our systematic literature review was to determine whether the new techniques have led to improvements in both outcomes and toxicities. Methods: A computerized search was performed by MEDLINE and the Cochrane database. The studies reported data from patients treated with carbon ion radiotherapy (CIRT), intensity-modulated photon radiotherapy (IMRT), and stereotactic radiotherapy (SBRT). Results: Seven publications of the 126 titles/abstracts that emerged from our search met the inclusion criteria and presented outcomes of 230 patients. OS was reported with rates of 90.0% and 73.0% at 1 and 2 years, respectively; LC was 89.0% and 71.6% at 1 and 2 years after re-RT, respectively. Toxicity data vary widely, with emphasis on acute and chronic gastrointestinal and urogenital toxicity, even with modern techniques. Conclusion: data on toxicity and outcomes of re-RT for LRRC with new technologies are promising compared with 3D techniques. Comparative studies are needed to define the best technique, also in relation to the site of recurrence.

## 1. Introduction

Rectal cancer (RC) ranks eighth worldwide in incidence of neoplasia, with an age-standardised rate of 1.73 per 100,000 persons/year. GLOBOCAN 2020 estimates that there are 0.7 million new cases of rectal cancer, and this number is expected to increase to 1.16 million in 2040 [1,2]. Neoadjuvant chemoradiation (CRT), total mesorectal excision, and adjuvant therapy have helped to reduce local failure of rectal cancer, but despite this, the incidence of locally recurrent rectal cancer (LRRC) is still 4–8% [3,4].

Although the rate of local recurrence after multimodality treatment, including neoadjuvant CRT, surgery, and adjuvant CRT, is low, 81% of all recurrences occur in the irradiation field or at its margins, and a total of 78% of field recurrences occur in the lower pelvic and presacral regions [5].

To date, the treatment of choice for LRRC is surgery with radical margins (R0). In cases where this is not possible, radiotherapy (RT) in combination with or without chemotherapy (CHT) is a viable alternative that may lead the patient to radical surgery. Resection of LRRC is more difficult due to the altered and diverse anatomy of the organs and critical structures in the pelvis. In addition, the presence of fibrosis after treatment makes surgery more difficult and decreases the chance of an R0 margin [6,7,8,9].

For this reason, re-irradiation (re-RT) may play a role in increasing the rate of radical resection or in the definitive treatment of inoperable patients [10,11,12].

The trouble concerning re-RT in this group of patients is related both in terms of the received dose of the organs at risk (OARs) and the time elapsed between the two irradiations. There are not enough studies on dose constraints for OARs, so radiation oncologists do not have clear guidelines on the doses that can be administered to avoid acute and late side effects [13]. Administering a suboptimal dose for fear of side effects can result in failure to control the disease or leave patients permanently inoperable [14,15,16].

There is no doubt that great progress has been made in the radiation treatment of patients with LRRC. Modern techniques and daily imaging monitoring allow highly conformal treatments to be delivered to the target site while avoiding OARs. Nevertheless, there are few studies on the use of these techniques in rectal cancer recurrence re-RT.

Previous literature reviews aimed to evaluate the efficacy of re-RT and determine the optimal treatment for LRRC [17,18]. They concluded that re-RT had favorable survival outcomes when combined with surgery and showed good oncologic and palliative efficacy with or without surgery. Unfortunately, most of these studies used 3D techniques. Nowadays, most RT centers have and use advanced technologies, so the literature data based on 3D techniques are no longer reliable for determining the doses that can be used to avoid the side effects of re-RT in this patient population [13].

In addition, radiation therapy is increasingly moving toward the use of new technologies: Carbon ion RT (CIRT), proton therapy (PBR), and MR-Linac-guided adaptive RT.

The aim of our systematic literature review is to examine studies in which these techniques have been used to understand whether they have an impact on oncological outcomes and toxicities in patients treated with re-RT for LRRC.

## 2. Materials and Methods

This systematic review was based on Preferred Reporting Items for Systematic Reviews and Meta-analysis (PRISMA) guidelines [19]. We systematically searched the MEDLINE and Cochrane databases through December 2022, with an updated search in April 2023, for the following terms: ((re irradiation) OR (re-RT) OR (re-irradiation)) AND ((rectal cancer) OR (rectal neoplasm) OR (local recurrent rectal cancer) OR (recurrent rectal cancer) OR (locally recurrent rectal cancer)). No publication date restrictions were applied. This study has not been registered. 

We included studies published in the English language with at least 10 patients with LRRC treated with re-RT with or without concomitant CHT. Prospective, retrospective, and randomized controlled trials were included. Case reports and reviews were excluded. Studies must have used more than 50% IMRT/Volumetric Modulated Arc Therapy (VMAT) or stereotactic body RT (SBRT) techniques. Studies that used CIRT, PBR, or SBRT or studies conducted with MR-linac were eligible for our review. Studies were included if they reported at least one of the following objectives: primary objectives, including overall survival (OS) and local control (LC), and secondary objectives, including grade 3 complications.

Initial screening was performed using titles to filter out studies that were duplicated in databases or that were not clinical trials, such as reviews, letters, editorials, case reports, and consensus guidelines. Studies that did not involve LRRC patients and brachytherapy studies were also excluded.

We performed a second screening based on abstracts and excluded studies that were not clinical trials, studies with fewer than 10 patients, studies with irrelevant subjects, non-English language studies, and abstracts only.

We then performed a full-text review to identify studies that met the above inclusion criteria. In the case of multiple studies from a single institution, we included the studies with the largest number of patients with LRRC who received re-RT. The above screening processes were performed independently by two authors (EG, SB), and the final inclusion was confirmed by mutual agreement.

Data acquisition was performed by two independent authors. We collected all general information regarding authors, publication year, country, number of patients involved and age, study design, study period, patient population, inclusion criteria, patients excluded, treatment information, re-RT technique, previous RT dose (Gy), interval between RT and re-RT, re-RT total dose and fractionations, cumulative dose, concomitant CHT, and percentage of patients that underwent surgery. We reported acute complications, defined as complications occurring within 3 months after re-RT or those described as acute complications in the relevant study. Late complications were defined as complications occurring after 3 months from re-RT or those described as late complications in the relevant study. Toxicities were classified into five categories: gastrointestinal (GI), genitourinary (GU), neuropathy, pain, and infection. Oncological outcomes included OS, progression-free survival (PFS), described as no progression of disease in the treatment area or outside the treatment field, and LC.

For missing numerical data, PFS, OS, or LC rates were estimated from descriptive graphs, if available. The information regarding tumor control was collected and analyzed as rates at specific time points (e.g., 1-, 2-, 3-, and 5-year LC rates), taking into account the possible recurrence or regrowth after re-irradiation.

If discrepancies in the information were found by the two independent researchers, the differences were resolved through discussion and repeated literature review.

## 3. Results

**Figure 1:**
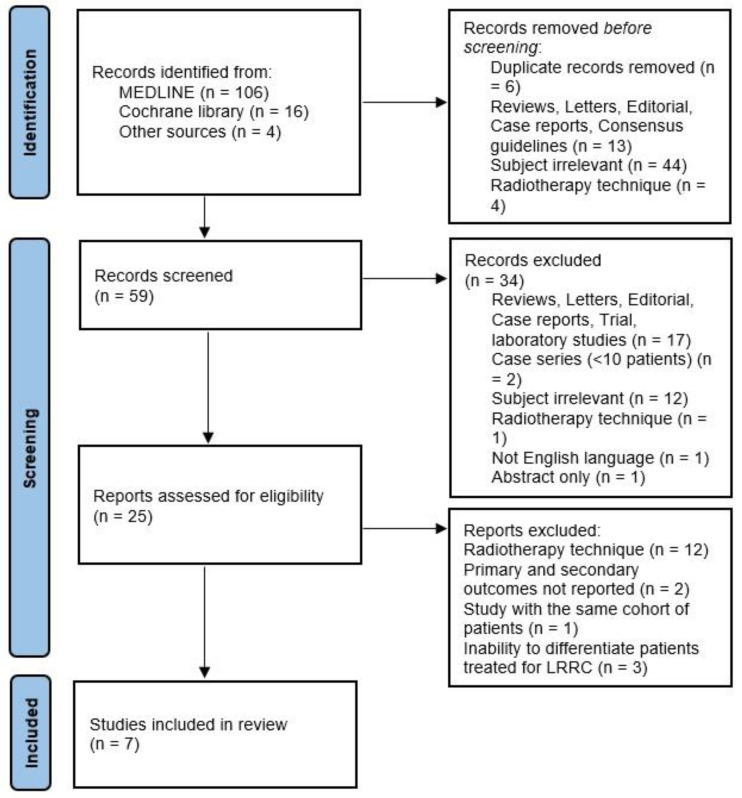
Search flow chart according to the PRISMA guidelines.

We identified 126 studies in MEDLINE and the Cochrane Library. After records were removed for title review, 59 studies underwent abstract review. A total of 67 did not meet the inclusion criteria: 17 were unsuitable types of publications (reviews, letters, editorials, case reports, trials, and laboratory studies), 12 were on irrelevant subjects, 2 had less than 10 patients, 1 was excluded for RT technique, 1 was not in the English language, and 1 was an abstract only. A full-text review was performed for 25 studies. A total of 12 studies were excluded for the RT technique; 1 study was excluded because multiple studies were published by a single institution, 2 studies were excluded because no outcomes of endpoints were provided for included patients, and finally, 3 studies were excluded because it was not possible to distinguish patients treated for LRRC in them. Overall, seven studies with eight cohorts of 230 patients were included in this review [20,21,22,23,24,25,26]. The study inclusion process is summarized in Figure 1.

The included papers were published from 2011 to 2022; six trials were retrospective studies [20,21,22,23,25,26], and one was a phase two study [24].

The analyzed patients were treated from 2003 to 2019 for previously irradiated LRRC.

Four papers specified exclusion criteria, which were metastatic patients or frail clinical conditions [20,21,22,24]. Table 1 reports the study and patient characteristics.

The Re-RT technique was CIRT in three papers [21,22,23], IMRT, SBRT, or Cyberknife in three papers [24,25,26], and one paper [20] considered both techniques. No studies were found on re-RT performed with MR linacs.

The median age calculated from all median ages reported in studies is 61 years old. The range is between 53 and 68 years old [20,21,22,23,24,25,26].

Previous RT dose was reported in all studies, and it ranged between 45 Gy and 50.4 Gy (median 50.2 Gy) [20,21,22,23,24,25,26]. The interval between first and second RT was reported in five studies [21,22,23,24,25], and it ranged between 22 and 65 months (median 47.4 months).

All papers [20,21,22,23,24,25,26] reported a total re-RT dose, with a median of 46.8 Gy (range 16.0–70.4 Gy). Dose fractionation was reported in all studies, ranging from 1.3 Gy BID to 12 Gy/fraction (median 4.4 Gy/fraction) [20,21,22,23,24,25,26].

The use of sequential CHT is reported in four studies [20,21,24,25]: two papers did not administer any CHT [21,25], one administered capecitabine or 5-fluorouracil-based for patients who have undergone re-RT with photons, while no patient who underwent CIRT received CHT [20]; in the last one [24], only 5-fluorouracil-based CHT was administered. Surgery was reported in four studies [20,21,22,24]; three of these reported 0% of patients initiated surgical treatment [21,22,24], and only one [20] reported the percentage of patients undergoing surgery, again for patients who have undergone re-RT with photons (while patients who underwent CIRT had no surgery before or after re-RT).

Only one study had a palliative intent [24]. 

Considering RT characteristics, all papers [20,21,22,23,24,25,26] reported the RT technique used. RT characteristics are summarized in Table 2.

Patients’ treatment positions were reported in five studies [22,23,24,25,26]: three papers treated them in a supine position [23,25,26], one in a prone position [22], and another [24] in both settings. 

Fractionation was reported in five studies and was daily in three studies [20,21,25]; one proposed bi-daily fractionation [24], and another 2-3 times per week [26]. 

Gross tumor volume (GTV) determination was described in five studies [21,22,23,24,25] and included the use of TC, MRI, and PET imaging. The clinical target volume (CTV) definition was explicated in five studies [20,21,22,23,26] and was generally GTV + 5–10 mm; only in one study was it identical to the GTV [26]. The planning target volume (PTV) definition was reported in six studies [20,21,22,23,24,26]. Dose constraints were reported extensively in three papers [20,21,24]. 

Regarding the size of the recurrence, three studies reported the average size of the disease in mm [20,21,23], and three studies reported the average volume in cm^3^ [22,25,26].

Six studies reported data on the site of recurrence [20,21,22,24,25,26]. Only one study included patients with extrapelvic metastases (4 patients out of a total of 22 patients) [24].

Outcomes are described in Table 3. All studies reported median follow up that ranged from 8 to 45.7 months (median 20.4 months). 

The Median OS was reported in five studies [20,21,23,24,25], and it ranged between 9.1 and 47.0 months, with a median value of 36.9 months. One-year and two-year OS were reported in six studies [20,21,22,24,25,26], with a median value of 90.0 (range 76.8–100%) and 73.0% (range 27.2–93.0%), respectively. Three-year OS was reported in four studies [20,21,22,25], with a median value of 61.0% (range 54.5–86.4%). Five-year OS was reported in three studies [20,21,26], with a median value of 49.0% (range 30.0–62.0%).

Six papers [20,21,22,23,25,26] reported results in terms of one-year LC. The median value was 89.0% (range 78.0–100.0%). Four papers [21,22,24,25] reported 2-year LC, with a median value of 71.60% (range 52.0–93.7%). Four papers [20,21,25,26] registered 3-year LC, with a median value of 69.0% (range (range 30.0–87.0%). Two papers registered 5-year LC, with a median value of 62.0% (range 55.0–70.0%) [20,21]. 

Data regarding PFS were reported in four studies [21,22,24,25]: median 1-year PFS was 65.6% (range 58.0–80.2%), and median 2-year PFS was 39.5% (range 10.7–68.7%).

The overall rate of the G3 acute toxicity rate was registered in six studies [21,22,23,24,25,26], and it ranged from 0% to 22.7%. Six papers recorded acute G3 GI toxicity [21,22,23,24,25,26], and it ranged from 0% to 13.6%. Acute G3 GU toxicity was reported in six studies [21,22,23,24,25,26] ranging from 0% to 5.5%. Five studies registered acute G3 neuropathy [21,22,23,25,26], and it ranged from 0 to 5.5%. Acute G3 pain and infection were reported in three studies [21,22,23] ranging from 0 to 2.6% and 0 to 6.5%, respectively.

Overall, the G3 late toxicity rate was registered in five studies [20,21,22,23,24], and it ranged from 0% to 37.7%. Five papers reported late G3 GI toxicity [20,21,22,23,24], and it ranged from 0% to 19.3%. Late G3 GU toxicity was recorded in five studies [20,21,22,23,24], and it ranged from 0% to 13.0%. Three studies registered late G3 neuropathy [21,22,23] ranging from 0 to 5.2%. Late G3 pain and infection were reported in three studies [21,22,23] ranging from 0 to 2.6% and 0 to 16.9%, respectively.

## 4. Discussion

This literature review confirms the role of RT in the re-RT of LRRC and that new technologies have an impact on outcomes and toxicity.

Over the years, the interest in re-RT of LRRC has grown exponentially. The difficulty of planning treatment considering the dose previously received by patients, the establishment of dose limits for OARs, the combination with concomitant CHT, and the definition of a dose have led to a growing interest in this topic, resulting in several studies and literature reviews. At the same time, highly conformal techniques have been developed to circumvent the problems of dose distribution of 3D techniques. 

Previously, several literature reviews have shown that re-RT is possible with good results in terms of local disease control and symptom relief [17,18,27]. 

A previous systematic review by Guren et al. supported the use of re-RT for LRRC, followed by radical resection when possible and hyperfractionation to reduce late toxicities [17]. A recent systematic review and meta-analysis by Lee et al. confirmed the oncologic efficacy of re-RT in LRRC and a higher survival rate with concurrent surgery but with a higher risk of late toxicities. The median OS in this study was more than 2 years [18]. Both studies showed that re-RT was also effective in the palliative setting. These reviews were mainly based on studies in which 3D techniques were used [17,18]. Regarding CIRT, the systematic review by Venkatesulu et al. reports interesting results and describes CIRT as a promising new technology when re-irradiation is required [27]

A recent Italian study published by the Italian Association of Radiation and Clinical Oncology for Gastrointestinal Tumors (AIRO-GI) showed that most Italian centers have advanced technologies such as VMAT/IMRT/SBRT and daily image monitoring, which have led to breakthroughs in dose and fractionation [13].

To date, this is the first systematic literature review of re-RT for LRRC to include only highly conformed techniques (VMAT/IMRT) and the first to include not only SBRT treatments but also treatments with CIRT.

In our systematic literature review, four studies used CIRT [20,21,22,23]. In two of them, PFS was measured at a median rate of 61.1% and 39.5% at 1 and 2 years, respectively [21,22]. OS was measured at a rate of 97.0%, 76.2%, and 76.2 at 1, 2, and 3 years in three studies, respectively [20,21,22]. The median local control at 1 year was 85.0% in four studies [20,21,22,23], and two studies reported a median local control at 2 years of 63.5% [21,22].

Regarding photons, the median PFS was 73.6% and 39.7% at 1 and 2 years, respectively, in two studies [24,25], while the median OS was 87.4% and 62.4% at 1 and 2 years in four studies [20,24,25,26] and 56.9% at 3 years in two studies [20,25]. In three studies, it was possible to calculate the median LC, which was 90.9% at 1 year [20,25,26], whereas it was 80.9% at 2 years [25,26], and 44% at 3 years [20,25,26]. 

Moreover, the RT intent in the Cai et al. study was palliative, and this may have influenced the lowest OS and PFS, especially at two years [24].

Our systematic review shows treatment results that seem to be superior to those reported by Lee et al., who found a pooled 1-, 2-, and 3-year rate OS of 76.1%, 49.1%, and 38.3%, respectively, and a 1-, 2-, and 3-year rate LC with pooled rates of 76.3%, 51.9%, and 46.4%, respectively [18].

The toxicity reported by the studies is extremely exogenous and exhibits wide variability. Two studies reported the absence of acute and chronic G3 toxicity in patients treated with CIRT [22,23], whereas reported acute toxicity in the study by Yamada et al. was 10.4%; chronic toxicity was 37.7%, with GI (11.7%) and infectious (16.9%) side effects predominating [21]. Chung et al. also reported data on late GI toxicity (5.7%), indicating that half of the patients treated with CIRT with G3 or higher toxicity received CHT after re-RT [20]. In contrast, among patients treated with photons, only the study by DeFoe et al. reported no acute toxicity after re-RT with Cyberknife [26], whereas only two studies reported acute G3 toxicity ranging from 16.5% to 22.7% [24,25]; chronic GI toxicity was reported in two studies ranging from 13.6 to 19.3% [20,24].

As for the systematic review and meta-analysis by Lee et al., for acute complications, the overall grade was 3, a pooled rate of 11.7%. For late complications, the pooled rate was 25.2%. Regarding GI, GU, and skin and soft tissue complications, rates of 13%, 2%, and 9% were found for acute toxicity and 13%, 9%, and 16% for late toxicity, respectively [18].

There is no way yet to determine whether the latest RT techniques result in a reduction in toxicity, although we affirm that with the latest technology, it has been possible to increase the dose to the target with acceptable side effects. To limit the dose to OARs, selected patients had received spacer implantation prior to CIRT or had an omental flap or polytetrafluoroethylene (PTFE) prosthesis inserted through open surgery to limit the dose to the bladder or bowel [20,21,22]. Moreover, one of the methods to reduce the side effects is to increase the number of fractions. In the study by Dagoglu et al., the selection of SBRT dose fractionation was based on the relationship of the recurrence to the dose-limiting structures, primarily the bowel. When the bowel was close to the target, five fractions were used, and this scheme was also used when there was no clear plane between the bowel or sacral plexus and the recurrence [25]. Regarding dose constraints, the Yamada et al. study set dose constraints for D2cc of the intestine and bladder at 60 Gy (RBE) and 70 Gy (RBE), respectively, when combined with the dose distribution of the previous RT [21].

A viable alternative to the presence of mobile OARs close to the target to be irradiated can be MR-guided radiotherapy. This technique allows daily identification of the target and OARs and dose modulation according to physiological changes in the anatomy [28,29]. 

Currently, there are no studies reporting the use of MR-linac in LRRC, but studies of adaptive radiotherapy in locally advanced rectal cancer are ongoing, so a role for this advanced technology in LRRC may also be envisioned [30]. 

It would have been interesting to have a comparison between the re-RT group and the re-RT plus surgery group. In our review, only the study conducted by Chung et al. considered patients who underwent surgery. In this study, 11 patients (30%) underwent resection before or after re-RT. However, they performed a multivariate analysis of factors associated with severe late toxicity, and surgery before or after re-RT was not statistically significant (*p* = 0.491) [20]. Surely, studies are needed to better investigate this aspect, leveraging a larger sample of patients.

The complexity of the treatment of LRRC led to the creation of additional therapeutic strategies developed over the years to meet the requirements of personalized treatment. In this scenario, the combined use of surgery and intraoperative RT also offers a promising approach to improve local control of the disease [31].

In addition, new biological discoveries may influence new drugs in the future that, in combination with RT, could improve curative options. Studies are also being developed to complement non-pharmacologic therapies [32].

The limitations of this literature review should be considered. First, the included studies are few and heterogeneous, in which patients were treated with different techniques, making any comparison difficult. Second, most of the included studies are retrospective. In addition, it is practically difficult to evaluate complications; patients who underwent re-irradiation for LRRC usually have a short life expectancy and poor compliance; moreover, most of the included studies described complications independently. Further prospective studies are needed to understand the role of modern technology in the re-RT of LRRC and whether it is possible to find a correlation between the site of recurrence and the best technique to use. A prospective observational multicenter study was recently designed to evaluate whether the combination of total neoadjuvant therapy (TNT) with re-RT in LRRC patients could lead to a better LC rate. The results of this study will hopefully provide a full understanding of the benefit of re-RT in different clinical settings of relapse [33].

## 5. Conclusions

Treatment of LRRC is certainly still an open challenge. The multidisciplinary team meeting certainly reflects the first step to be taken to identify operable recurrences in the first instance and to identify patients who could benefit from pre-operative or radical re-RT. 

Modern photon RT techniques (VMAT/IMRT/SBRT) have brought a breakthrough in improving dose conformity. They allow higher doses of radiation to be delivered to the tumor while minimizing the dose to surrounding normal tissue. This can lead to better tumor control rates and fewer side effects. 

Using advanced technologies such as CIRT and MR-guided RT offers potential advantages that can improve treatment accuracy and outcomes, but both are technologies that are not available in all RT centers. Networking between RT centers equipped with different technologies could be a key step in personalizing treatment.

With the aim of investigating whether the combination of total neoadjuvant therapy (TNT) with re-RT in LRRC patients could lead to a better LC rate and to evaluate the role of CIRT followed by CHT in patients considered inoperable, a new multicenter observational study has been started [33].

New prospective data on integrating different anti-cancer treatments, the RT technique, the observed toxicity, and the outcomes are needed to better tailor treatments.

## Figures and Tables

**Table 1:** Patient and study characteristics.

Author	N° Patients	Country	Year	Study Design	Study Period	Patient Population	Re-RT Technique	Age, (Range) Years	Previous RT Dose (Range), Gy	Interval between RT (Range), mo	Re-RT Total Dose, Gy	Re-RT Fx. Dose, Gy	CTx. Rate (%) (Agent)	Surgery
Chung SY	35	Japan-Korea	2022	R	2005–2019	LRRC	CIRT	62 (37–76)	50 (20–66)	NR	70.4 Gy (RBE) 101.38 Gy in BED10	4.4 Gy [RBE]	Not administered	0%
	31						29% 3D RT, 71% IMRT or Cyberknife	60 (35–87)	50.4 (45–60)	NR	50 Gy (range 25–62.5 Gy) 60 Gy in BED10		68% *	23% After, 13% Before re-RT
Yamada S	77	Japan	2022	R	2005–2017	LLRC	CIRT	60 (37–76)	50 (20–74)	50 (13–157)	70.4 Gy (RBE)	4.4 Gy [RBE]	Not administered	0%
Barcellini A	14	Italy	2020	R		LRRC	CIRT	58.5 (34–78)	45 (45–76)	65 (14–139)	60 Gy RBE (35–76.8)	3 Gy RBE (3–4.8)	NR	0%
Habermehl D	19	Germany	2014	R	2010–2013	Unresectable LRRC	CIRT	62 (14–76)	50.4 (50.4–60.4)	47.4 (17–110)	36 to 51 Gy (RBE)	3 Gy (RBE).	NR	NR
Cai G.	22	China	2014	Phase II	2007–2012	Unresectable LRRC	IMRT	53 (40–68)	48.6 (36–62)	30 (18–93)	39	1.3 BID	81.8% (5-FU based)	0%
Dagoglu N.	18	Turkey	2015	R	2006–2012	Pelvic RRC or CC	Cyberknife	68 (32–93)	50.4 (25–100.4)	22 (15–336)	25 (24–40)	5	Not administered	NR
DeFoe S.G.	14	USA	2011	R	2003–2008	Presacral RRC	Cyberknife	65.5 (42–77)	50.4 (20–81)	NR	16 (12–36)	12	NR	NR

Abbreviation: BID, twice daily, CIRT, carbon ion radiotherapy, CTx, chemotherapy, Fx, fractions, Gy, Gray, IMRT, intensity-modulated radiotherapy, LRRC, local recurrent rectal cancer, mo, month, MRI, magnetic resonance imaging, N, number of patients, NR, not reported, R, retrospective, RBE, radiobiological equivalent, RRC, recurrent rectal cancer, Re-RT, re-irradiation, RT, radiotherapy, 3D, three-dimensional, and 5-FU, 5-fluorouracil. * Capecitabine, 5-fluorouracil/leucovorin, and 5-fluorouracil/leucovorin/oxaliplatin.

**Table 2:** Treatment characteristics.

Author	ReRT Technique	Position	Fractionation	GTV	CTV	PTV	Recurred Tumor Size/Volume mm/cm^3^ (Range)	Site of Recurrence Number (%)	Dose Constraints
Chung S.Y.	CIRT	NR	Daily, 4 days a week	NR	GTV + 5 mm	NR	25 (15-80) mm	Non-presacral, regional, nodal 17 (49%) Presacral 18 (51%)	Dose constraints of D2cc for the bowel and bladder were 44 Gy (RBE) and 50 Gy (RBE) in 16 fractions, respectively
	3D - IMRT or CyberKnife	NR	25 Fx (range 3–33)	NR	NR	GTV of the recurred tumor plus a 0.5–3-cm margin	30 (10-70) mm	Non-presacral, regional, nodal 22 (71%) Presacral 9 (29%)	NR
Yamada S.	CIRT	NR	Daily, 4 days a week	Macroscopic tumor visible on CT, MRI, and PET imaging	GTV + 5 mm	CTV + 3-5 mm	40 (14-110) mm	Presacral 29 (43.3%) Side wall 23 (34.3%) Perineal 15 (22.4%)	D2cc of the intestine and bladder were set at 50 Gy (RBE) and 60 Gy (RBE) in 16 fractions, and at 60 Gy (RBE) and 70 Gy (RBE) when combined with the dose distribution of the previous RT
Barcellini A.	CIRT	Prone	NR	Area of Contrast Enhancement on T1-Weighted MRI Images.	GTV + 5–10 mm	CTV + 3–10 mm	154.63 (7.2–359.9) Volume cm^3^	Presacral 10 (72%) Perineal 1 (7%) Perianal 1 (7%) Pre-Coccygeal 2 (14%)	NR
Habermehl D.	CIRT	Supine	NR	Macroscopic tumor visible in T1-weighted MRI	GTV + 5–10 mm	CTV 3–10 mm based on individual anatomical factors	58 (12–112) mm	NR	NR
Cai G.	IMRT	Prone or supine	BID 5 days per week	Defined from CT, MRI, and/or PET/CT	NR	GTV + 2–3 cm	NR	Perirectal region 5 (20%) Presacral region 7 (28%) Internal iliac nodal region 7 (28%) Perineum 5 (20%) External iliac nodal region 1 (4%).	Small bowel: no more than 180 cc above 20 Gy, no more than 65 cc above 30 Gy, and maximum dose less than 40 Gy; Femoral heads: no more than40% volume above 25 Gy, no more than 25% volume above 30 Gy, and maximum dose less than 40 Gy; Bladder: no more than 40% volume above 25 Gy, no more than 15% volume above 30 Gy, and maximum dose less than 40 Gy
Dagoglu N.	Cyberknife	Supine	Daily	CT images	NR	NR	90.1 cc (36.8–1029.4) volume cm^3^	Presacral 5 (23.8%) Pelvic side wall 12 (57.1%) Central pelvis 2 (9.5%) Presacral + Pelvic side wall 1 (4.8%) Pubic area 1 (4.8%)	Protocol from institution
DeFoe S.G.	Cyberknife	Supine	2–3 times per week or single dose	NR	Identical to GTV	GTV + 5 mm	52.5 (19–110) volume cm^3^	Presacral 100%	NR

Abbreviation: BID, twice daily, CIRT, carbon ion radiotherapy, CT, computer tomography, CTV, clinical target volume, Fx, fractions, GTV, gross tumor volume, IMRT, intensity-modulated radiotherapy, MRI, magnetic resonance imaging, NR, not reported, PET, positron emission tomography, PTV, planning target volume, RBE, radiobiological equivalent, Re-RT, re-irradiation, RT, radiotherapy, and 3D, three-dimensional.

**Table 3:** Outcomes.

Author	Re-RT Technique	Follow up (Range), Months	Progression-Free Survival (PFS)					Overall Survival (OS)					Local Control (LC)				
			Median (months)	1-year PFS	2-year PFS	3-year PFS	5-year PFS	Median (months)	1-year OS	2-year OS	3-year OS	5-year OS	Median (months)	1-year LC	2-year LC	3-year LC	5-year LC
Chung S.Y.	CIRT	45.7 (7–148.4)	NR	NR	NR	NR	NR	Not achieved	97%	93%	86.4%	62%	NR	94%	NR	87%	70%
	3D—IMRT or Cyberknife	22.8 (7.2–148.4)	NR	NR	NR	NR	NR	36.9	88.9%	59%	54.5%	30%	NR	89%	NR	44%	55%
Yamada S.	CIRT	45 (7–159)	14	58%	36%	33%	25%	47	90%	73%	61%	38%	NR	85%	75%	69%	62%
Barcellini A.	CIRT	18	m-PFS 14.4 (2–40)	64.3%	43%	NR	NR	NR	100%	76.2%	76.2%	NR	14.5 (2.4–49.5)	78%	52%	NR	NR
Habermehl D.	CIRT	8	NR	NR	NR	NR	NR	9.1	NR	NR	NR	NR	20.6 *	85%	NR	NR	NR
Cai G.	IMRT	17 (2–59)	NR	67%	10.7%	NR	NR	19	85.9%	27.2%	NR	NR	14	NR	NR	NR	NR
Dagoglu N.	IMRT	38 (6–36)	38	80.2%	68.7%	61.1%	NR	40	76.8%	65.9%	59.3%	NR	NR	100%	93.7%	85.9%	NR
DeFoe S.G.	cyberknife	16.5 (6–69)	NR	NR	NR	NR	NR	NR	90%	78.8%	NR	60%	NR	90.9%	68.2%	30%	NR

Abbreviation: CIRT, carbon ion radiotherapy, IMRT, intensity-modulated radiotherapy, LC, local control, m-PFS, metastases-free survival, NR, not reported, PFS, progression-free survival, OS, overall survival, 3D, three-dimensional, and * estimated local progression.

## Data Availability

Not applicable.
